# Metabolic syndrome and sexual function in postmenopausal women

**DOI:** 10.1590/2359-3997000000194

**Published:** 2016-08-23

**Authors:** Kathiussa Dombek, Emille Joana Medeiros Capistrano, Ana Carolina Carioca Costa, Lizanka Paola Figueiredo Marinheiro

**Affiliations:** 1 Departamento de Endocrinologia, Ginecologia e Obstetrícia Instituto Nacional de Saúde da Mulher, da Criança e do Adolescente Fernandes Figueira Fundação Oswaldo Cruz Rio de Janeiro RJ Brasil Departamento de Endocrinologia, Ginecologia e Obstetrícia, Instituto Nacional de Saúde da Mulher, da Criança e do Adolescente Fernandes Figueira, Fundação Oswaldo Cruz (IFF/Fiocruz), Rio de Janeiro, RJ, Brasil; 2 Departamento de Estatística Instituto Nacional de Saúde da Mulher, da Criança e do Adolescente Fernandes Figueira Fundação Oswaldo Cruz Rio de Janeiro RJ Brasil Departamento de Estatística, Instituto Nacional de Saúde da Mulher, da Criança e do Adolescente Fernandes Figueira, Fundação Oswaldo Cruz (IFF/Fiocruz), Rio de Janeiro, RJ, Brasil

**Keywords:** Metabolic syndrome, female sexual function, menopause, postmenopausal

## Abstract

**Objective:**

The purpose of this study was to evaluate whether female sexual dysfunction (FSD) is associated with metabolic syndrome (MS) and to identify factors that contribute to FSD in postmenopausal women.

**Subjects and methods:**

This was a cross-sectional study in 111 sexually active women aged 45-65 years. We applied the Female Sexual Function Index (FSFI) to evaluate the participant’s sexual function and a structured questionnaire to collect demographic, socioeconomic, clinical, anthropometric, and laboratory data.

**Results:**

The prevalences of MS and FSD were 68.5% and 70.3%, respectively. After logistic regression analysis, we identified the following variables associated with FSD: married status (prevalence ratio [PR] 1.69, 95% confidence interval [95% CI] 1.16-2.47, p < 0.01), 6-10 years elapsed since menopause (PR 1.60, 95% CI 1.22-2.09, p < 0.01), occurrence of climacteric symptoms (PR 1.01, 95% CI 1.00-1.02, p = 0.03), and history of sexual abuse (PR 1.40, 95% CI 1.12-1.73, p < 0.01).

**Conclusion:**

We found a high prevalence of MS and FSD, but no association between both. Married status, time elapsed since menopause, climacteric symptoms, and history of sexual abuse emerged as factors associated with FSD on multivariate analysis.

## INTRODUCTION

A woman’s sexual health is associated with several psychological and interpersonal factors, and may be affected by aging and metabolic changes. In this context, female sexual dysfunction (FSD) is a multifactorial and multidimensional biological problem (1). The concept of FSD includes the occurrence of difficulty in any phase of the sexual response cycle or pain associated with intercourse (2).

Menopause is a transitional life phase in which a woman undergoes hormonal, physical, psychological, and social adjustments. Previous studies have demonstrated that menopause may negatively influence sexuality, leading to an important dysfunctional condition and affecting quality of life (3,4).

The decrease in estrogen levels associated with menopause may lead to changes in lipid profile, increase in body weight, and accumulation of abdominal fat. A recent report addressed in the Princeton III Consensus suggests an association between female sexual function with cardiovascular and metabolic diseases (5).

During the climacteric phase, a woman’s cardiovascular risk profile undergoes changes characterized by the onset or worsening of some risk factors such as central obesity, hypertension, and dyslipidemia. These factors, in addition to hyperglycemia and insulin resistance, constitute the concept of metabolic syndrome (MS) (6).

Obesity, hypertension, dyslipidemia, and type 2 diabetes, which are conditions frequently present in individuals with MS, are considered risk factors for atherosclerosis and endothelial dysfunction. In FSD, endothelial dysfunction impairs tissue oxygenation and causes subsequent functional and structural damage to the female genital tract (7). A decrease in pelvic blood flow secondary to atherosclerotic disease leads to fibrosis of the vaginal wall and clitoral smooth muscle, eventually resulting in vaginal dryness and dyspareunia (8).

A study has shown the occurrence of low sexual function scores in premenopausal women evaluated with the Female Sexual Function Index (FSFI). Decreases in FSFI total scores were proportional to an increase in the number of MS components (9). Another study in postmenopausal women also found an association between MS and low sexual function scores (10). In contrast, Kim and cols., while studying middle-aged women, found no association between MS and most of the components of sexual function (11). Following the same line, a study in climacteric women by Politano and cols. found no association between MS and decreased sexual function, except for increased age which was associated with a decreased sexual function (12).

Among the various factors involved with female sexual function, MS seems to be an emergent concern, due in part to its elevated global prevalence. However, it is yet not known whether the effects of MS are significant enough to lead to FSD (11); in fact, data from the literature are still conflicting regarding the association between MS and FSD.

Both conditions are multifactorial, and their diagnoses depend on the characteristics of the studied population and the criteria adopted for diagnosis. The duration and severity of the MS also seem to have an effect on a woman’s sexuality; however, it is still unclear how severe the MS must be to exert this effect (11,12).

The purpose of the present study was to evaluate whether FSD is associated with MS and identify the factors that contribute to FSD in postmenopausal women.

## SUBJECTS AND METHODS

The study population in this observational, cross-sectional study comprised postmenopausal women attending the outpatient clinic of the Department of General Gynecology at the Fernandes Figueira National Institute for Women, Children, and Youth Health/IFF (Rio de Janeiro, Brazil) between August and December 2013.

In order to calculate the sample size, we used information from the study by Martelli and cols. (10) who evaluated the occurrence of FSD in postmenopausal women using the FSFI questionnaire. These authors found a prevalence of FSD of 68% in patients with MS, compared with 41% in those without MS. Based on that, we estimated in our study a sample size of 200 women. The sample size necessary to detect a difference in FSD prevalence was calculated at a power of 80% and a significance level (α) of 0.05. Since the sample size obtained for each FSFI domain was smaller than that calculated for all FSFI domains as a whole, we calculated the sample sizes at a lower power (56.89%) for the domain lubrication and at a higher power (95.81%) for the domain arousal.

The inclusion criteria comprised women aged between 45 and 65 years, with amenorrhea for longer than 12 months, serum follicle-stimulating hormone (FSH) level ≥ 40 mIU/mL (13), and reporting sexual activity within the last 4 weeks from the date of the interview. The exclusion criteria were the use of hormone replacement therapy (HRT) in the past year, past or current chemotherapy or radiation therapy due to cancer, pelvic surgery for bilateral oophorectomy, premature menopause, neurological disease, type 1 diabetes, thyroid disease, hyperprolactinemia, and homosexual relationship.

During the study period, a total of 416 postmenopausal women aged between 45 and 65 years visited the outpatient clinic and were invited to participate in the study. Of all women, 40 refused to participate, 46 had a history of cancer, 53 were on HRT, 24 had reached menopause prematurely, 37 had undergone bilateral oophorectomy, and two had neurological diseases. Of the remaining 214 women, two were excluded for failing to draw blood and 13 for presenting an FSH value ≤ 40 mIU/mL. Among the remaining 199 women, only 111 reported sexual activity within the prior 4 weeks from the study and were included in the analysis.

The project was approved by the Research Ethics Committee (REC) at the Fernandes Figueira Institute under protocol number CAAE 03498812.7.0000.5269 and REC certificate of approval number 359.174. All participants signed an informed consent form.

A structured questionnaire with closed questions was applied to each participant to identify demographic, socioeconomic, and clinical variables. The participants’ schooling level was evaluated by the number of years of complete study. The household *per capita* income was considered as the sum of all monthly earnings of the family members, and the result was categorized in numbers of minimum wages in Brazilian currency (Brazilian real [R$]).

We used the International Consultation on Incontinence Questionnaire – Short Form (ICIQ-SF) to evaluate the occurrence of urinary incontinence (UI). The ICIQ-SF is composed of four questions that evaluate the frequency, severity, and impact of UI. The scores of the total result of questions that evaluate severity range from 0 to 21 points (14). Women with scores ≥ 8 were considered symptomatic for UI (15).

Symptoms related to the climacteric phase were evaluated with the Blatt-Kupperman Menopausal Index (BKMI), which is a global quantitative evaluation of the occurrence of such symptoms. The symptomatology is evaluated with 11 questions regarding hot flashes, arrhythmias, dizziness, headache, paresthesia, tingling sensation, arthralgia and myalgia (classified as somatic symptoms), as well as fatigue, nervousness, and sadness (these last three symptoms are categorized as psychological symptoms). The higher the score, the more symptomatic the women were (16). For the present analysis, we used the mean total score of the questionnaire.

The participants’ sexual function was evaluated with the FSFI, a validated questionnaire that assesses the woman’s sexual response and quality of life over the previous 4 weeks (2,17). This questionnaire is constituted by 19 questions about the domains of sexual response: desire and subjective arousal, lubrication, orgasm, satisfaction, and pain or discomfort. Individual scores are obtained by the sum of the items that constitute each domain and are multiplied by the factor of this domain, resulting in the weighted score. The final score (which may range from a minimum of two to a maximum of 36) is obtained by the sum of the weighted scores in each domain. The cutoff value for the score that determines the occurrence of FSD is 26.5, with higher scores indicating better sexuality (17).

Two trained investigators applied the questionnaires and collected anthropometric measurements in a private room.

To establish the diagnosis of MS, we used the criteria defined by the Joint Scientific Statement for Harmonizing the Metabolic Syndrome issued by the International Diabetes Federation (IDF); American Heart Association; National Heart, Lung, and Blood Institute; World Heart Federation; International Atherosclerosis Society; and International Association for the Study of Obesity. We considered as having MS those participants who presented three of the following criteria: waist circumference ≥ 88 cm (as established for Brazilian women) (18), serum triglyceride level ≥ 150 mg/dL, serum high-density lipoprotein cholesterol (HDL-C) level ≤ 50 mg/dL, systolic blood pressure ≥ 130 mmHg and/or diastolic blood pressure ≥ 85 mmHg, and fasting blood glucose level ≥ 100 mg/dL. Use of medications and treatment for high blood pressure, diabetes, hypertriglyceridemia, and low HDL-C values were also considered in the diagnosis of MS since they are also part of the syndrome’s criteria (6).

Blood pressure was evaluated in the participant’s non-dominant arm using a mercury sphygmomanometer accurate to 2 mmHg while the participant remained seated after resting for at least 10 minutes. The participants’ weights were measured with a digital scale (Leader, Model LD1050) and represented in kilograms, and their heights were measured using a Wiso^®^ stadiometer 200 cm long and accurate to 0.05 cm.

The participants’ body mass index (BMI) was calculated by the weight divided by the square of the height and was represented in kg/m^2^. The BMI results were categorized according to the classification determined by the World Health Organization for adults (19). The patients were categorized as eutrophic when having a BMI between 18.5-24.9 kg/m^2^, overweight when between 25.0-29.9 kg/m^2^, and obese when ≥ 30.0 kg/m^2^.

Blood samples were collected after fasting for determination of levels of glucose, HDL-C, triglycerides, C-reactive protein (CRP), free thyroxine, thyroid-stimulating hormone, FSH, estradiol, and prolactin. Blood tests were evaluated by enzyme-linked fluorescence (ELFA) and immunofluorometric assays using the kit VIDAS^®^ (bioMérieux). An immunonephelometry assay was used to evaluate the serum concentrations of CRP using the CardioPhase® hsCRP reagent and a Siemens BN II equipment. According to the results of high-sensitive CRP (expressed in mg/dL), patients were classified as low risk (when < 0.1 mg/dL), intermediate risk (when between 0.1 and 0.3 mg/dL), and high risk (when > 0.3 mg/dL) (20).

### Statistical analysis

The results of the statistical analyses for continuous variables with normal distribution are presented as mean and standard deviation. When normality was not observed, the results are expressed as median and minimum and maximum values. The Shapiro-Wilk test was used to verify the normality of the data. Bivariate analyses were performed to identify factors potentially associated with MS. Pearson’s chi-square test measured the statistical significance of the association between MS and categorical variables. Fisher’s exact test was applied in cases with at least one expected frequency below five. To compare continuous measures, Student’s *t*-test was used for variables with normal distribution and the Mann-Whitney test for those without normal distribution. Poisson regression models with robust variance estimation were employed to determine factors associated with sexual dysfunction. The variance inflation factor was used to detect possible multicollinearity. Univariate regression analyses were carried out, and variables with a *p* value below 0.20 were included in a multiple regression model. The significance level adopted to define the factors statistically associated with sexual dysfunction in the multiple regression model was 5%. The magnitude of the association between explanatory and outcome variables was quantified as prevalence ratio (PR) and corresponding 95% confidence intervals (95% CI). The software used for the analysis of the data were SPSS, version 20, and R, version 3.0.2.

## RESULTS

Among the 111 women evaluated, the average age was 55.9 ± 4.8 years and 78.4% were married. The median duration of school education was 8 years and the mean household *per capita* income was R$ 600. Of all participants, 83.8% were multipara, the mean age at menopause was 48.3 years, 11.7% were smokers, 27.9% consumed alcohol regularly, 56.8% were sedentary, 57.7% used antihypertensive drugs, 23.4% used hypoglycemic drugs, and 16.2% used hypolipidemic drugs. A total of 68.5% of the participants had a diagnosis of MS (Table 1).

**Table 1:** Sociodemographic, clinical, anthropometric, and metabolic characteristics according to the presence or absence of metabolic syndrome (MS)

Variable	Metabolic syndrome			*P* value

	Total (n = 111)	Absent (n = 35)	Present (n = 76)	
Age (years)	56.0 (45-65)	55.5 (45-64)	57.0 (48-65)	**0.02**
Marital status				
Married	87 (78.4%)	27 (31.0%)	60 (69.0%)	1.00
Not married	24 (21.6%)	8 (33.3%)	16 (66.7%)	
Schooling years	8 (0-17)	8 (2-14)	8 (0-17)	0.22
Number of pregnancies				
Nulliparous	5 (4.5%)	4 (80.0%)	1 (20.0%)	**0.02**
Primiparous	13 (11.7%)	6 (46.2%)	7 (53.8%)	
Multiparous	93 (83.8%)	25 (26.9%)	68 (73.1%)	
Age at menopause	48.3 (±4.54)	47.9 (±3.70)	48.4 (±4.90)	0.60
Duration of menopause (years)				
1 to 5	50 (45.0%)	17 (34.0%)	33 (66.0%)	0.57
6 to 10	28 (25.2%)	10 (35.7%)	18 (64.3%)	
More than 10	33 (29.7%)	8 (24.2%)	25 (75.8%)	
Blatt-Kupperman Menopausal Index	22.7 (±12.83)	22.3 (±12.23)	22.9 (±13.18)	0.81
Smoker				
Yes	13 (11.7%)	4 (30.8%)	9 (69.2%)	0.91
No	61 (55.0%)	18 (29.5%)	43 (70.5%)	
Former	37 (33.3%)	13 (35.1%)	24 (64.9%)	
Alcohol consumption				0.65
Yes	31 (27.9%)	11 (35.5%)	20 (64.5%)	
No	80 (72.1%)	24 (33.8%)	56 (70.0%)	
Physical activity				
Active	48 (43.2%)	15 (31.2%)	33 (68.8%)	1.00
Sedentary	63 (56.8%)	20 (31.7%)	43 (68.3%)	
Body mass index				
Eutrophic	23 (20.7%)	15 (65.2%)	8 (34.8%)	**< 0.01**
Overweight	43 (38.7%)	14 (32.6%)	29 (67.4%)	
Obese	45 (40.5%)	6 (13.3%)	39 (86.7%)	
Waist circumference				
Normal	23 (20.7%)	16 (69.6%)	7 (30.4%)	**< 0.01**
Increased (≥ 88 cm)	88 (79.3%)	19 (21.6%)	69 (78.4%)	
Systolic blood pressure				
Normal	64 (57.7%)	28 (43.8%)	36 (56.2%)	**< 0.01**
Elevated (≥ 130 mmHg)	47 (42.3%)	7 (14.9%)	40 (85.1%)	
Diastolic blood pressure				
Normal	64 (57.7%)	29 (45.3%)	35 (54.7%)	**< 0.01**
Elevated (≥ 85 mmHg)	47 (42.3%)	6 (12.8%)	41 (87.2%)	
Blood glucose				
Elevated (≥ 100 mg/dL)	55 (49.5%)	4 (7.3%)	51 (92.7%)	**< 0.01**
Normal	56 (50.5%)	31 (55.4%)	25 (44.6%)	
High-density lipoprotein cholesterol				
Low (< 50 mg/dL)	61 (55.0%)	12 (20.0%)	48 (80.0%)	**< 0.01**
Normal	50 (45.0%)	23 (45.1%)	28 (54.9%)	
Triglycerides				
Increased (≥ 150 mg/dL)	45 (40.5%)	6 (13.3%)	39 (86.7%)	**< 0.01**
Normal	66 (59.5%)	29 (43.9%)	37 (56.1%)	
C-reactive protein				
Low < 0.1 mg/dL	27 (25.0%)	11 (40.7%)	16 (59.3%)	**< 0.01**
Moderate 0.1–0.3 mg/dL	42 (38.9%)	19 (45.2%)	23 (54.8%)	
High > 0.3 mg/dL	39 (36.1%)	5 (12.8%)	34 (87.2%)	

Tests: Student’s t-test for equality of means, Mann-Whitney U test, Pearson’s chi-square test, and Fisher’s exact test.

On univariate analysis, the following variables were potentially associated with FDS: married status (*p* = 0.02), *per capita* income (*p* = 0.05), vaginal childbirth (*p* = 0.02), time elapsed since menopause (*p* < 0.01), BKMI value (*p* = 0.02), prior bladder surgery (*p* < 0.01), alcohol consumption (*p* < 0.01), and history of sexual abuse (*p* < 0.01).

Regarding the FSFI, we analyzed the total scores and the scores in each domain of the questionnaire. The median FSFI score in the overall cohort was 23.9, which is below the cutoff score of 26.5 determined for the questionnaire (Table 2). To evaluate the relationship between the components of MS and FSD, we analyzed the total FSFI scores according to the number of MS components, but we found no significant differences. After a multiple regression analysis, we identified the following variables associated with FSD: marital status, time elapsed since menopause, climacteric symptoms, and history of sexual abuse (Table 3).

**Table 2:** Total score and scores in each domain of the Female Sexual Function Index (FSFI) questionnaire according to the presence and absence of metabolic syndrome

Domain		Metabolic syndrome		*P* value

	Total (n = 111)	Absent (n = 35)	Present (n = 76)	
FSFI	23.9 [8.8-34.9]	22.8 [10.0-32.5]	24.8 [8.8-34.8]	0.25
Desire	3.0 [1.2-6.0]	2.4 [1.2-4.8]	3.0 [1.2-6.0]	0.12
Arousal	3.6 [1.2-6.0]	3.3 [1.2-5.7]	3.9 [1.2-6.0]	0.07
Lubrication	4.2 [1.2-6.0]	3.6 [1.2-6.0]	4.2 [1.2-6.0]	0.62
Orgasm	4.0 [1.2-6.0]	4.0 [1.2-6.0]	4.2 [1.2-6.0]	0.75
Satisfaction	4.8 [0.8-6.0]	4.8 [1.2-6.0]	4.8 [0.8-6.0]	0.63
Pain	4.0 [0.0-6.0]	3.6 [1.2-6.0]	4.4 [0.0-6.0]	0.47

Test: Mann-Whitney U.

**Table 3:** Multiple regression model of variables associated with risk of sexual dysfunction

Variable		Adjusted prevalence ratio (95% confidence interval)	*P* value
Marital status			
Married		1.69 (1.16-2.47)	**< 0.01**
Not married		1.00	---
*Income per capita*		0.99 (0.99-1.00)	0.07
Number of vaginal births		0.98 (0.89-1.07)	0.61
Number of cesarean births		0.94 (0.80-1.10)	0.41
Duration of menopause			
1 to 5 years		1.00	---
6 to 10 years		1.60 (1.22-2.09)	**< 0.01**
> 10 years		0.94 (0.69-1.28)	0.67
Blatt-Kupperman Menopausal Index		1.01 (1.00-1.02)	**0.03**
Bladder surgery			
Yes		1.02 (0.75-1.37)	0.92
No		1.00	---
Smoker			
Yes		1.04 (0.56-1.97)	0.89
No		1.00	---
Former		1.16 (0.91-1.49)	0.23
Alcohol consumption			
Yes		0.74 (0.50-1.10)	0.14
No		1.00	---
History of sexual abuse			
Yes		1.40 (1.12-1.73)	**< 0.01**
No		1.00	---
C-reactive protein			
Low		1.00	---
Moderate		1.07 (0.82-1.40)	0.60
High		0.89 (0.66-1.20)	0.45
Waist circumference (≥ 88 cm)			
Increased		0.83 (0.65-1.07)	0.14
Normal		1.00	
Blood pressure (≥ 130/≥ 85 mmHg)			
Elevated		1.02 (0.82-1.26)	0.87
Normal		1.00	
Triglycerides (≥ 150 mg/dL)			
Elevated		1.09 (0.86-1.37)	0.47
Normal		1.00	
High-density lipoprotein cholesterol (< 50 mg/dL)			
Low		1.31 (0.97-1.78)	0.08
Normal		1.00	

Poisson’s regression model.

## DISCUSSION

In the present study, we found no association between MS and FSD. The FSD rate in women with MS was not higher than that in the group of women without MS. Similarly, none of the FSFI domains were associated with MS, showing that the sexual function of the participants was not significantly impaired by the occurrence of MS.

To establish the diagnosis of MS in our study, we used the criteria defined by the Joint Scientific Statement for Harmonizing the Metabolic Syndrome (6) and identified an MS prevalence of 68.5% among postmenopausal women. Another cross-sectional study that has evaluated the sexual function of climacteric women with MS found a prevalence of 62.1% when using the criteria defined by the IDF. In contrast, in the study by Ponholzer and cols., who also adopted the IDF criteria, the prevalence of MS in postmenopausal women was 32.6% (7).

Studies carried out in populations from different countries have revealed a high prevalence of MS, with rates oscillating from 8.0% to 24.0% in men and 7.0% to 46.0% in women (21). In postmenopausal women, MS rates have been reported to vary between 22.0% and 69.0% (22). Overall, the MS rates vary according to the diagnostic criteria adopted in the study and characteristics of the observed population, including sex, age, and ethnicity of the participants and associated morbidities.

We chose the FSFI questionnaire because this instrument is easily administered in women of a broad age group, including postmenopausal women, but also because it is widely used in international research, as reported in several studies (9,11,10,23). The FSFI was developed as a brief self-report instrument to assess the main dimensions of the female sexual function. The questionnaire was designed to evaluate the female sexual response and quality of life in clinical and epidemiological studies (17), and it is in line with new models of female sexuality (2). The current standard cutoff value of the total FSFI score to diagnose sexual dysfunction is 26.5. Therefore, women presenting scores equal to or below 26.5 are considered to have sexual dysfunction (24).

Previous studies have demonstrated a close relationship between MS and male sexual dysfunction, especially related to erectile dysfunction (25,26), and more recent discussions have attempted to show this association between MS and FSD (9,10).

One of the first studies to find a positive association between MS and FSD was published by Esposito and cols. in the evaluation of premenopausal women. These authors observed a decrease in the FSFI score in women with MS when compared with controls. Although the authors adopted the FSFI questionnaire to evaluate sexual function, they used another scale to determine the occurrence of FSD (9). Another study in postmenopausal women also found a higher prevalence of FSD in women with MS when compared with controls, demonstrating a possible association between FSD and MS. This study also showed that high levels of triglycerides are associated with a higher risk of FSD (10).

Ponholzer and cols., in a cohort study including premenopausal and postmenopausal women, reported an association in premenopausal women of MS and FSD, restricted to the desire component of the sexual function. However, MS had no effect on any of the sexual function components in postmenopausal women when comparing those with and without MS. This result may have differed because these authors used a questionnaire validated for the evaluation of women’s sexual health (7).

Kim’s and cols., while studying sexual function in middle-aged women, noticed that MS had little influence on their participants’ sexuality (11). Another cross-sectional study carried out in climacteric women evaluated the relationship between decreased sexual function and MS and also found no association between FSD and MS, with the exception of older age, which was associated with decreased sexual function (12).

These differences between studies can occur due to several factors such as differences in study design, population ethnicity, diagnostic criteria and cutoff points for both FSD and MS. Another question that must be considered is that FSD can be different according to the severity of the components of the MS (11). In our study, we were unable to evaluate the association between FSD and severity of MS due to the small number of participants.

Based on the pathophysiology of MS, this condition may affect the female sexual function due to its association with vascular inflammation and endothelial dysfunction, which can impair vessel oxygenation and reduce the blood supply to the pelvis (27). However, vascular inflammation and endothelial dysfunction are not frequently observed in the initial phase of MS. Following this hypothesis, the female sexual function would only be impaired in more severe or chronic cases of MS. This contrasts with the observations in men, in whom sexual dysfunction is intimately associated with cardiovascular diseases and MS in the very beginning of the disease (28).

Inflammatory biomarkers are important tools to monitor the progress of endothelial dysfunction. The inflammatory marker CRP is associated with cardiovascular morbidity in the long-term and has a stronger prognostic value regarding cardiovascular events than other biomarkers such as homocysteine and lipoprotein. Moreover, it has already been shown that patients presenting four or five MS components are at increased risk of developing cardiovascular disease (29). Based on this information, we chose to use CRP to verify a possible association between MS and FSD. Esposito and cols. found an inverse relationship between FSFI scores and CRP levels (9). However, the present study found no association between CRP and FSD.

On multivariate analysis, we found that married status was a risk factor for FSD. Another study that has reported similar results demonstrated that married status was a stronger risk factor for FSD and that the partner had an important influence on the woman’s sexuality.

According to our findings, women who had reached menopause 6-10 years before the study had a higher risk of FSD. Some studies have demonstrated that the increase in age and time elapsed since menopause may be associated with FSD (30), since women with more time elapsed since menopause may be more susceptible to suffering the consequences of the decline in hormone levels, which increase their chances of having vaginal atrophy, urinary tract infections, UI, and FSD (8).

Although FSD increases with age, it seems that the distress associated with the loss of sexual desire is minimized with age (31). This was observed in a study by Graziottin, who found that the distress associated with FSD is more prevalent in younger women (32).

The biological components of sex and sexuality change substantially with aging in terms of intensity and quality of sexual response. However, some women who are more experienced at this phase of life have fewer conflicts regarding their sexuality. This allows them to seek new ways to exercise their sexuality, motivated by their developed wisdom, better knowledge of their bodies, and maturity (13). This may explain why women who reached menopause more than 10 years before our study have a lower risk of FSD than those who did so between 6 and 10 years before.

The results of our study show that the occurrence of climacteric symptoms, evaluated by the BKMI had a negative influence on the participants’ sexual function. This finding is aligned with that by De Lorenzi and Saciloto (30) in a study evaluating the frequency of sexual activity in postmenopausal women. Another study, also on the influence of climacteric symptoms on sexual function in middle-aged women, has demonstrated that the greater the severity of the symptoms, the higher the chance of the woman of presenting FSD (4).

During menopause, women present higher vasomotor, psychological, and urogenital symptoms associated with hypoestrogenism. The decrease in estrogen levels results in a reduction of pelvic support and lubrication of urogenital tissues, causing pain and difficulty during sexual activity. However, sexuality is not influenced only by hormonal factors; it is also associated with the emotional status of the woman, the quality of her relationship with her partner, and the environment in which she lives (33).

Other important aspects that interfere with the capacity of sexual response are psychological factors. Low self-esteem, anxiety, past traumatic experiences, sexual violence, sexual abuse during childhood, and rape create a negative impact on a woman’s sexuality (34). In our study, a history of sexual abuse was associated with the highest risk of FSD. A dysfunctional condition may appear due to organic causes, but it will very often be aggravated by an emotional and psychosocial event (35).

Some limitations of our study must be considered in the interpretation of our results. Considering that the study had a cross-sectional design, we are unable to infer a cause-and-effect relationship or evaluate the onset of FSD and MS. The women who participated in our study may not represent those in the general population, given that they were recruited from an outpatient clinic. Another factor that must be considered is the small number of participants in our study. Although more than 400 women visited the outpatient clinic, only 111 satisfied all inclusion criteria of the study.

Further longitudinal studies are required to evaluate the temporality and causality between FSD and MS and its components, and the factors predisposing to FSD. This should be done taking into account the fact that both FSD and MS are multifactorial conditions; therefore, the impact of confounding factors must be considered in the analysis, including psychological issues and relationship with the partner, among others.

In conclusion, we found no association between MS and FSD, or influence of metabolic risk factors on the domains of female sexual function. The women in our study population had high prevalences of FSD and MS, the latter attributing a high cardiovascular risk profile. Multivariate analysis evidenced that the factors associated with FSD were married status, time elapsed since menopause, climacteric symptoms, and history of sexual abuse.

The fact that FSD has been found to be associated with different factors support the concept that the female sexuality is a very complex and multifactorial subject that requires further studies. Offering monitoring and support for both women and men regarding aging and its complications can help prevent MS and improve sexual function and quality of life.
